# Seismic localization of elephant rumbles as a monitoring approach

**DOI:** 10.1098/rsif.2021.0264

**Published:** 2021-07-14

**Authors:** Michael Reinwald, Ben Moseley, Alexandre Szenicer, Tarje Nissen-Meyer, Sandy Oduor, Fritz Vollrath, Andrew Markham, Beth Mortimer

**Affiliations:** ^1^ Department of Zoology, University of Oxford, Oxford, UK; ^2^ Department of Computer Science, University of Oxford, Oxford, UK; ^3^ Department of Earth Sciences, University of Oxford, Oxford, UK; ^4^ Mpala Research Center, Nanyuki, Kenya; ^5^ Save the Elephants, Marula Manor, Karen, Nairobi, Kenya

**Keywords:** animal behaviour, behavioural ecology, wildlife monitoring, vibrational communication, *Loxodonta africana*

## Abstract

African elephants (*Loxodonta africana*) are sentient and intelligent animals that use a variety of vocalizations to greet, warn or communicate with each other. Their low-frequency rumbles propagate through the air as well as through the ground and the physical properties of both media cause differences in frequency filtering and propagation distances of the respective wave. However, it is not well understood how each mode contributes to the animals’ abilities to detect these rumbles and extract behavioural or spatial information. In this study, we recorded seismic and co-generated acoustic rumbles in Kenya and compared their potential use to localize the vocalizing animal using the same multi-lateration algorithms. For our experimental set-up, seismic localization has higher accuracy than acoustic, and bimodal localization does not improve results. We conclude that seismic rumbles can be used to remotely monitor and even decipher elephant social interactions, presenting us with a tool for far-reaching, non-intrusive and surprisingly informative wildlife monitoring.

## 1. Background

African elephants (*Loxodonta africana*) use a wide variety of vocalizations to communicate with other elephants, from trumpets and snorts to infrasonic rumbles [1]. These vocalizations, or calls, convey a variety of meanings to aid in their social organization, including greetings, warnings about imminent threats, communicating movement or advertising reproductive state to the other sex [2].

The low-frequency rumbles are especially interesting as the vocalization generates both an acoustic and a seismic component simultaneously [3,4]. An elephant’s inner ear is particularly well suited to detect these low-frequency sounds as they have an enlarged malleus [5]. Behavioural responses of elephants to seismic playback of rumbles have shown that the seismic component alone is sufficient to elicit a behavioural response [6]. Elephants are thought to detect seismic vibrations either through sensors embedded in the skin on their feet and/or through bone conduction of vibrations to the inner ear [7]. The possession of a sphincter-like muscle in the outer ear of elephants, as well as behavioural adaptations such as freezing and leaning behaviour, is thought to aid in the detection of the seismic component of elephant rumbles [8]. It is an open question whether the information contained in acoustic and seismic components of rumbles is redundant or whether it provides different or complementary information, which is likely to be dependent on changing environmental factors [9,10].

In order to gain useful information, elephants show remarkable sophistication in signal processing of detected rumbles [8,11], as shown by their behavioural responses, including the ability to accurately categorize and localize calls. Elephants often need to determine where a rumble is coming from, in terms of its direction and sometimes distance, to respond appropriately. For example, walking towards female elephant oestrus calls is particularly important for male elephants, as shown with acoustic playback experiments [12]. The mechanism of localizing seismic vibrations is unknown, where differences in seismic waves would need to be detected by sensors in different feet or ears, which could be maximized by turning perpendicular to the source [6]. To estimate the azimuth (i.e. horizontal direction) of an acoustic sound source, elephants probably use the time shift between a sound arriving at the two ears, called the interaural phase difference (IPD) [13,14]. For distance estimation, there are multiple acoustic cues available (intensity, direct-to-reverberant energy ratio or spectral cues) and their reliability can vary according to the properties of the environment, the direction of the sound source and the stimulus [15]. Difference in intensity is not thought to be used for acoustic localization because the dimensions of the animal’s head (diameter of ∼1 m) are mostly smaller than half the wavelength of the propagating wave for frequencies smaller than 170 Hz (assuming a wavespeed of approx. 340 m s^−1^), thus it is unlikely that there will be a detectable intensity difference across ears [16]. Instead, the auditory system can determine phase delays without confusion and ambiguity [17,18]. It has also been suggested that differences in time of arrival between acoustic and seismic waves could be used to estimate distance to the sender of a rumble [6,11,19]; since the acoustic and seismic wavespeeds differ, in theory distance to the source could be determined if the two wave types can be detected separately [20].

Humans too can record the seismic and acoustic waves generated by elephants in order to determine their location. Sensor systems have been suggested for this purpose, using single or multi-modal systems with the potential to give real-time information to respond to poaching threats or prevent human–elephant conflict [21–26]. Techniques that can accurately monitor elephants in this way will be crucial tools for fundamental and applied research alike.

Here, we use seismic signals generated by vocalizing wild elephants to constrain their positions, which is compared with localization using the co-generated acoustic component. We calculate localization errors and infer the spatial information content of the respective signals using the same methods for seismic and acoustic signals. Typically, localization routines (in an artificial sense) involve signal processing, such as time/frequency filtering, noise reduction or signal enhancement, detection of the desired signal and position estimation [27].

Raw signals collected during deployment usually contain a cacophony of biological, geological and anthropogenic vibrations and noise [26]. Noise reduction can be used to filter out unwanted signals and emphasize the desired signal. In addition, frequency filtering [28,29] cancels out signals above or below a certain frequency, or in a certain frequency range, which is helpful if the frequency content of the source signals is well known. Other methods of signal enhancement are spectrogram filtering using a time/frequency structure tensor [21] and edge detection of the target signal [30].

Detecting the target signal in a (pre-)processed recording often requires manual identification of relevant events. Rhinehart *et al*. [27] reviewed recent studies on acoustic localization of terrestrial wildlife and highlighted that greater than 50% of studies used manual sound detection routines and less than 10% had fully automated routines. The main reason for this is that the accuracy of manual detection routines is considered high compared with automated methods [31]; however, it is very time consuming and practically unfeasible for very large datasets. Importantly, only fully automated techniques could possibly be used for the purpose of operational monitoring, ideally in near real time. New deep-learning-based detection and classification techniques promise high accuracy with much faster processing than the human eye [32,33].

Signals recorded in multiple spatial locations can be used to localize an event/source. One of the most common position estimation algorithms is the time-difference-of-arrival (TDOA) technique, in which it is assumed that the wave radiates out spherically from the source. It uses the different arrival times of the source signal, e.g. a vocalizing animal, measured at all receivers, to calculate possible locations of the source [27], much like mammals use IPD at two ears to estimate source azimuth. Differences in arrival times are usually calculated using cross-correlation [34,35] of the signals in either time-amplitude or time-frequency representation. These two representations are prone to the same errors, such as synchronization lags between the devices [36,37] and wrong position information of the devices, as well as fluctuations in the wavespeed [38,39], errors in the calculations of the arrival times/angles [40–42] or distances that are too large between source and receiver [43]. It is assumed that the neural mechanisms used by animals to calculate IPDs are similar to cross-correlation of the two signals [44].

In this study, we developed deterministic and Bayesian (probabilistic) TDOA algorithms to localize African elephants using seismic and acoustic recordings of their rumbles. We compare the efficacy of localization with seismic or acoustic data, providing a novel means of monitoring vocalizing elephants. Given the complexity and frequency differences of the heterogeneous terrain through which seismic waves propagate, along with the frequency filtering differences between seismic and acoustic propagation, it could be argued that acoustic waves might have advantages in terms of accuracy of localization of rumbles and potential applications, yet here we aimed to explicitly test that hypothesis. We therefore tested if seismic waves alone are sufficient to localize elephant rumbles within a practical degree of accuracy and compared localization accuracy with the same framework using the co-generated acoustic signals, as well as a bi-modal framework, i.e. using both. Furthermore, we aimed to determine the suitability of localization using seismic recording as a tool to monitor elephants and study their communication, specifically their low-frequency rumbles. Overall, this study provided us with crucial insight into the suitability and accuracy of seismic signals for localizing elephants with high precision to monitor their behaviour in complex or unknown terrains, representing an important step in better understanding their multi-modal means of communication.

## 2. Methods

### 2.1. Data collection

Data were collected in February and March 2019 at Mpala Ranch on the Laikipia Plateau in central Kenya (0°27'19.26"N, 36°50'33.02"E) for a continuous duration of three weeks. This region around the Ewaso Ng’iro River and its tributaries is characterized by arid and semi-arid climate, consisting mostly of savannahs and woodlands. Mpala Ranch covers an area of approximately 48 000 acres and is estimated to have over 500 avian and around 100 mammalian species, including large populations of resident and migrant elephants.

**Figure 1.**
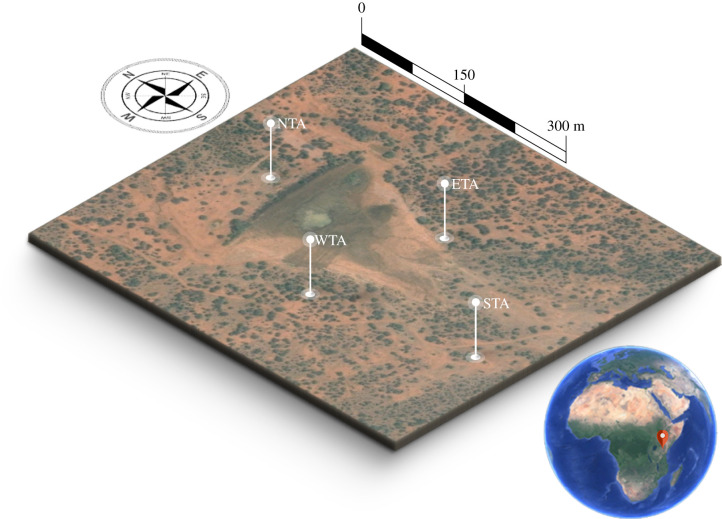
Overview of the study area: the study was carried out using four seismic/acoustic stations (coded ETA,NTA,WTA,STA) surrounding a watering hole at Mpala Ranch, located on the Laikipia Plateau in central Kenya. Satellite image data taken from Google Maps.

For this particular study, we used data from four broadband seismometers (Guralp 6TD) and four acoustic sensors (custom-made) [35], co-located at four locations, and data from 13 motion-triggered camera traps (Reconyx, USA) at separate locations for partial visual coverage of the area. All devices surrounded a watering hole and were located within about 2 m elevation above the waterhole, which was obstructed by a dam (about 6–8 m height) at its northern edge (figure 1).

All seismic field sensors were buried about 70 cm deep in mostly dry-sand soil and were protected by a cage, recording ground motion in three orthogonal components with a sampling frequency of 200 Hz. All acoustic sensors were installed inside the cages, and recorded audio data on four channels, with a sampling frequency of 44.1 kHz and 16 bit digitization. For the purpose of this study, all four channels were stacked to increase signal strength. All instruments used GPS synchronization for accurate timing of signals. The seismic sensors have a flat frequency response between 0.03 Hz and 100 Hz in all components [45]. The microphones’ sensors (ICS 41350) have a flat frequency response between 100 Hz and 4 kHz with a roll-off in amplitude for lower frequencies, i.e. −2 dB for 50 Hz, −4 dB for 40 Hz, −10 dB for 20 Hz [46]. All sensor and camera data were locally stored onto hard drives. Seismic and acoustic data were stored in MSEED format (a simplified version of SEED—Standard for the Exchange of Earthquake Data [47]), and images were saved as JPG raster formats.

**Figure 2.**
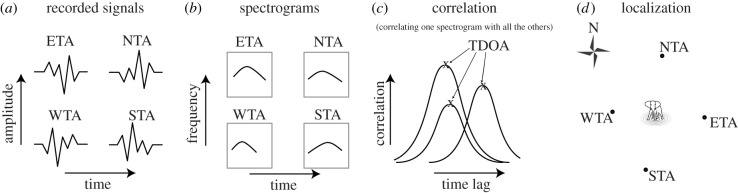
Illustration of data processing which applies to acoustic and seismic data: spectrograms (*b*) of the four recorded rumble signals (*a*) are used to calculate the TDOA between the station pairs, by correlating the spectrograms along the time axis (*c*). Calculated TDOAs are then used in the various localization frameworks to estimate the position of the elephant source (*d*).

We applied a sequence of data-processing steps to turn raw seismic and acoustic signals into a measurement that was robust for localization algorithms (figure 2). Details of the techniques are given in the following sections and electronic supplementary material.

### 2.2. Pre-processing of data

To detect time–frequency-dependent signals, such as rumble vocalizations, we applied a short-time Fourier transformation to the signals, to create two-dimensional spectrograms of the data (figure 2*a*,*b*). In such an image, the *x*-axis represents time, the *y*-axis represents frequency and the colour represents magnitude, or amplitude, of a frequency at a particular time. This calculation is typically done over certain windows of the signal, with overlapping windows ensuring sufficient resolution in time. Furthermore, we truncated high frequencies that did not include any signals.

Important factors that constitute the spectro-temporal structure of a sound are frequency contours and spectral peaks. To further enhance the contours of vocalizations in the spectrograms, we applied an automated image enhancement algorithm on all data. The idea, here, is to enhance vocalizations with sharp contours and spectral peaks along the frequency axis in the spectrogram, whereas homogeneous and isotropic signals, such as noise or other broadband signals, are attenuated. The detection of contours and peaks in spectrograms is similar to detecting edges and corners in images. A powerful method for the detection of such structures is the structure tensor of an image [48], which describes image gradients and which we applied to the spectrograms (see electronic supplementary material).

### 2.3. Event selection

The event selection process of our method also relied on manual detection of rumbles in all spectrograms. For this comparative study, we set the following requirements for signals to be considered:

— All four acoustic and all four seismic sensors recorded the event.
— All eight recordings showed a rumble (manual visual assessment of the spectrograms, looking for signals of 3–5 s duration with characteristic frequency modulation—first increasing then decreasing peak frequency between 20 Hz and 40 Hz).
— For all station pairs, there was good enough correlation between the signals, measured by the peak prominence of the correlation, i.e. the vertical distance between one single peak and its lowest contour line.

Regarding the first criterion, because of battery issues at one seismic station, the amount of time that all four stations could record was halved. Regarding the second criterion, we discovered an abundance of seismic rumbles in the data; however, most of them were not visible at all stations. This could be due to multiple sources of seismic vibration obfuscating the signals, as our chosen location was crowded with wildlife. For the third criterion, by setting a minimum value of all peak prominences in the correlation step, we minimized the chances of selecting an event that was not suitable for localization; however, it is possible that some events were missed. We used a peak-finding algorithm in the correlation curves and chose events based on the prominence of the detected peak, but not the actual correlation value due to the high noise in some of the data.

Following the above protocol, we isolated nine events for processing and subsequent deterministic and probabilistic localization (electronic supplementary material). Since camera traps were not recording continuously, nor were they covering all locations by the dam, they only provided images for visual ground truthing for one of the nine events. To further validate the accuracy of the seismic localization, we also include two additional events in electronic supplementary material, Events A1 and A2, figures S10–S13 and table S1 that did not explicitly meet the selection criteria; there was no clearly visible rumble on all four acoustic stations for both of them. However, owing to camera ground truthing in both cases, we can show successful localization of the rumbling elephants. We suggest that our event selection criteria are conservative and the presented method could work on data with lower signal-to-noise ratios across all signals.

### 2.4. Localization methods

**Figure 3.**
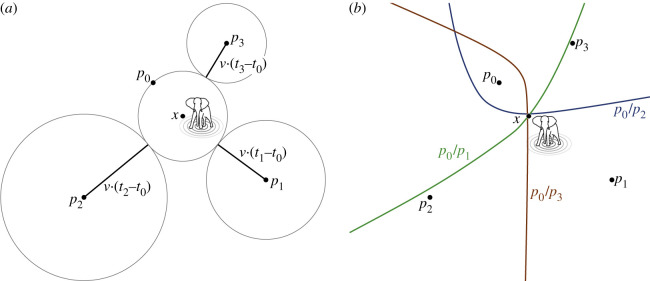
Illustration of the relationship between animal, receiver location, TDOAs and corresponding hyperbolas. (*a*) Position ***x*** is the animal’s location, each point ***p***_*i*_ is a station, *v* is the estimated wavespeed and *t*_*i*_ is the time at which the signal was recorded at ***p***_*i*_. The circles represent the times taken for the sound to travel from the centre along the length of the radius. (*b*) Each station pair and its respective hyperbola is highlighted in a different colour. The estimated location of the vocalizing animal is at the intersection of all hyperbolas, i.e. here position ***x***.

We considered all four stations to be in a two-dimensional horizontal plane. The signal (in our case a rumble vocalization) generated by the elephant at location ***x*** at time *t* was received by stations ***p***_*i*_ (*i* = 0,1,2,3) at time *t*_*i*_, respectively, depending on the speed of propagation *v* of the signal and the distance ||***x*** − ***p***_*i*_||, where the station that received the signal first was defined as ***p***_0_. We then know that the signal has travelled for (*t*_0_ − *t*) time and a distance of *v* · (*t*_0_ − *t*) = ||***x*** − ***p***_0_||, where *t* and ***x*** may not be known. The TDOA between ***p***_0_ and ***p***_*i*_ (*i* ≠ 0) can then be used with an estimate of the wavespeed *v* to calculate the difference in distance *v* · (*t*_*i*_ − *t*_0_) travelled by the signal between the time *t*_0_ (when station ***p***_0_ registered the signal) and time *t*_*i*_ (when station ***p***_*i*_ registered the same signal). Figure 3*a* shows the relationship between the location of the event (i.e. animal), the position of the stations and the relative TDOAs (*t*_*i*_ − *t*_0_) (for an arbitrary wavespeed *v*).

For each station pair ***p***_0_/***p***_*i*_, the calculated TDOA represents a two-dimensional hyperbola containing possible locations of ***x*** (for an illustrative example, see figure 3*b*). In theory, two equations (or three stations in total) are enough to narrow ***x*** down to at least two points, and with three equations (or four stations) to exactly one position, by either plotting the hyperbolas onto a map or, as in our case, solving the problem numerically (see electronic supplementary material).

We used and compared two distinct methods of localization: deterministic and probabilistic, calculating TDOAs between station pairs using cross-correlation of the spectrograms (time/frequency representations) of the respective signals (see electronic supplementary material). While amplitude-based cross-correlation could be more beneficial for real-time applications we are using spectrogram-based cross-correlation as it has given better results in our framework/analysis. In summary, both methods estimated the vocalizing animal’s positions and a corresponding wavespeed that best fits all of the calculated TDOA values. The deterministic method used the calculated TDOA values and a range of wavespeed guesses, whereas the probabilistic method incorporated uncertainties in the TDOA calculations and wavespeeds.

### 2.5. Localization accuracy/residual

We calculated the estimated error of the model (i.e. residual, unit: metres) in both methods by comparing the calculated TDOAs, i.e. calculated time delays from correlating the spectrogram pairs (see §2.4), with the estimated TDOAs (using the estimated location, its distance to the respective stations and the estimated wavespeed, we can estimate the time it takes the signal to travel to each station and, hence, estimate the corresponding TDOAs). In an ideal case, the localization output should give the same TDOA values for calculated and estimated outputs. The difference between the calculated TDOA and the estimated TDOA was averaged across station pairs and divided by the wavespeed estimate to give a measure of error, or conversely accuracy of the localization in metres. For each localization event we calculated the smallest location error over the solution samples proposed by each localization method.

## 3. Results

**Figure 4.**
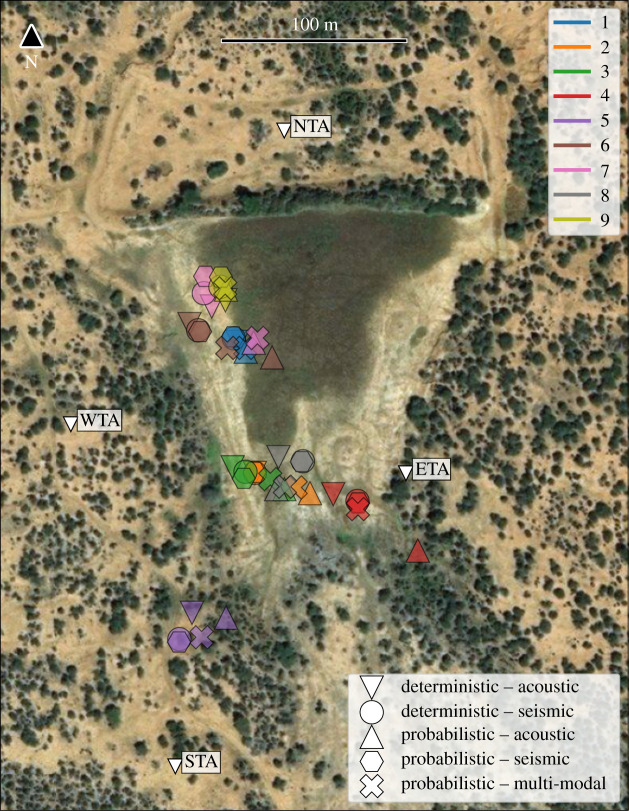
Summary of all estimated locations for the nine events that met our inclusion criteria: each event is shown in a different colour, with different icons for the various localization methods (i.e. deterministic, probabilistic) and data types (i.e. acoustic, seismic, multi-modal). Locations represent the position estimated by each method with the smallest residual error. Satellite image data taken from Google Maps.

We applied our data-processing steps and localization algorithms for both seismic and acoustic data from a selection of nine elephant rumbles that met our inclusion criteria, ensuring sufficient seismic and acoustic signal strength and correlation at all four stations. For each rumble, we calculated the ‘best’ location estimate and its corresponding residual, i.e. its associated error estimate, using the deterministic approach (on seismic and acoustic data) and the probabilistic approach (on seismic, acoustic and combined data) separately. Figure 4 plots the location estimates of each rumble on a map for all five data/approach combinations and table 1 gives the day, time and location residuals of each rumble event.

**Table 1.** Summary of estimated residuals: time and day of each event and minimum residual estimates. Residuals are shown in metres and the most reliable localizations (lowest residual error) for each rumble and localization method are shown in italics.

			smallest residual error (m)				
event number	day	time	deterministic acoustic	deterministic seismic	probabilistic acoustic	probabilistic seismic	probabilistic joint
1	23 February 2019	20:43:32	3.98	*0.02*	3.33	*0.57*	4.07
2	23 February 2019	21:01:47	4.19	*0.09*	3.86	*0.33*	11.79
3	23 February 2019	21:06:32	*1.03*	5.63	*1.01*	5.20	15.54
4	23 February 2019	21:44:12	2.24	*1.48*	1.76	*1.35*	6.69
5	24 February 2019	00:21:22	13.04	*4.06*	10.71	*3.24*	11.16
6	24 February 2019	17:42:30	0.45	*0.02*	0.56	*0.45*	14.53
7	1 March 2019	08:41:35	23.75	*23.21*	*15.38*	19.35	22.56
8	2 March 2019	10:06:50	*0.05*	1.63	*0.27*	1.47	8.01
9	6 March 2019	22:20:12	12.18	*10.10*	9.75	*8.06*	10.08
average			6.77	*5.14*	5.18	*4.45*	11.59
s.d.			7.98	*7.55*	*5.42*	6.16	5.50

### 3.1. Method validation

Our two independent methods show considerably precise localization of all elephant rumbles, as can be seen by an overlap of location and residual estimates of less than 25 m (figure 4 and table 1) with stations being up to 360 m apart. The independent methods also showed good agreement in the location of the elephant; locations (figure 4) and corresponding errors (table 1) were highly correlated between the two independent methods using either acoustic or seismic data (unimodal), with a Pearson correlation coefficient of 0.9898 and 0.9990 calculated between the residuals of the two methods for all rumbles for only acoustic and only seismic data, respectively. We verified the position of the rumbling elephant for one out of nine rumbles for which we had a camera trap image (electronic supplementary material, figure S1), further supporting the validity of the localization outcomes in this field case where no other ground truth reference was available.

### 3.2. Seismic versus acoustic localization

Both seismic and acoustic data alone were sufficient to localize the elephant that generated the rumble, with generally overlapping location estimates (figure 4). The average residual error for acoustic localization was 6.77 m (deterministic method) and 5.18 m (probabilistic method). By comparison, the average error for seismic localization was 5.14 m (deterministic method) and 4.45 m (probabilistic method). Seismic localization therefore caused a reduction in error of around 20% compared with acoustic localization (table 1). Six out of all nine rumbles showed smaller residual values for seismic localization in both localization frameworks (deterministic, probabilistic). Using both seismic and acoustic data bimodally caused a reduction in accuracy compared with using seismic or acoustic data alone; residual estimates of the joint method (i.e. using both seismic and acoustic data using the probabilistic method) were larger than the unimodal methods (mean: 11.59 m) and showed correlation values of 0.7540 and 0.5067 (compared with probabilistic seismic and acoustic residuals, respectively).

### 3.3. Example localization of one rumble

Going into more detail for one rumble allows us to demonstrate the processing steps and localization framework in more detail, highlighting the advantages, limitations and potential of this method to localize elephants. Here, we will demonstrate all steps for one selected rumble (event 8 in table 1), the event where location was verified using camera trap footage. Full outputs from the other selected rumbles can be found in the electronic supplementary material.

**Figure 5.**
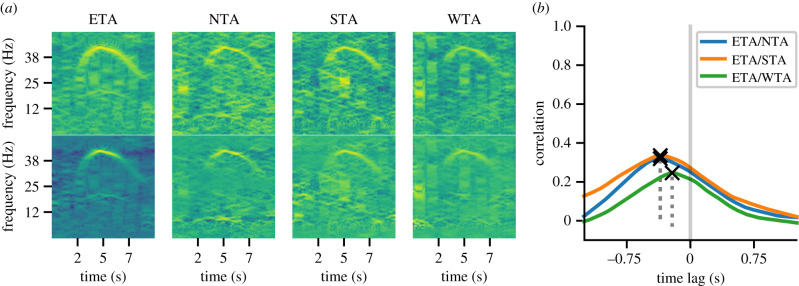
Seismic spectrograms and TDOA calculation. (*a*) Extracted seismic rumbles from the four stations before (top row) and after (bottom row) spectrogram enhancement. (*b*) Correlation curves for each station pair are shown by different colours. Corresponding prominences are shown by dashed lines and the maximum of each correlation, marked as an *x*, is the resulting TDOA value (in seconds).

**Figure 6.**
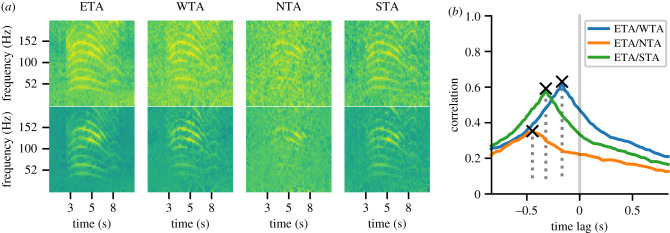
Acoustic spectrograms and TDOA calculation. (*a*) Extracted acoustic rumbles from the four stations before (top row) and after (bottom row) spectrogram enhancement. (*b*) Correlation curves for each station pair are shown by different colours. Corresponding prominences are shown by dashed lines and the maximum of each correlation, marked as an *x*, is the resulting TDOA value (in seconds).

Spectrograms for all four seismic and all four acoustic stations for event 8 are shown in figures 5*a* and 6*a*. By inspection of maximum intensity, the rumble was most pronounced at station ETA; hence, this station was chosen to be ***p***_0_, while the other three stations were ***p***_1/2/3_ in arbitrary order. The following analysis was done separately for acoustic and seismic signals: spectrograms were enhanced and correlated, respectively (for more details, including equations, see electronic supplementary material), resulting in three TDOA values (ETA/WTA, ETA/NTA, ETA/STA) and their respective prominences, a measure for correlation quality. An example of the three relative correlations, and the resulting time lags (TDOAs) and prominences, is shown in figures 5*b* and 6*b*.

**Figure 7.**
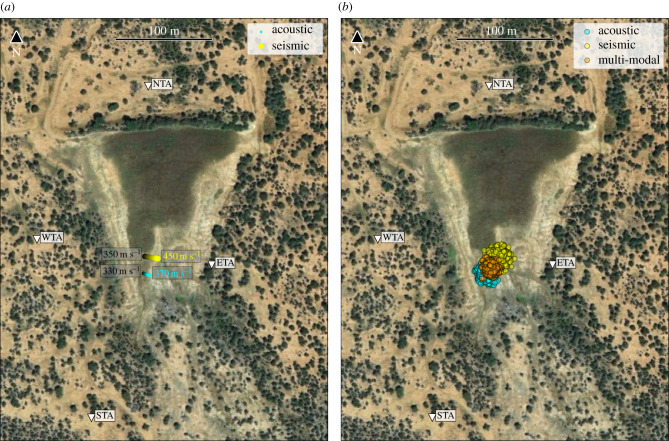
Localization results for event 8. (*a*) Deterministic location results with circle markers showing the estimated locations for different acoustic (cyan) and seismic (yellow) wavespeeds. (*b*) Probabilistic location results with circle markers showing the estimated location for each sample from the posterior distribution using acoustic data (cyan), seismic data (yellow) or both (multi-modal, orange). Satellite image data taken from Google Maps.

Localization results are shown in figure 7*a* (deterministic) and figure 7*b* (probabilistic). For both methods, the location and corresponding constant wavespeed were chosen out of all localization estimates (coloured dots in figure 7) that minimized the residual estimate. Both methods, deterministic and probabilistic, localized the rumble with a minimum residual error smaller than 2 m (table 1). The rumble event was captured by one of the camera traps, with the photo taken 10 s past the event (see electronic supplementary material, figure S1). This confirmed that elephants were present at the estimated time and position.

## 4. Discussion

In this study, we aimed to determine if seismic recordings of elephant rumbles could be used to locate African elephants and determine whether these recordings could be used as a tool to localize elephants in order to monitor them and study the use of seismic waves in elephant communication. We focused our efforts on a quantitative comparison of two independent localization methods and their accuracy, based on a residual error of each location estimate, to provide validation of the outputs of the approach. Furthermore, we compared the use of seismic and acoustic recordings to localize elephant rumbles, including the use of uni- and bimodal data. Our results highlight the advantages of using seismic data to monitor and study African elephants.

For our selected rumbles, seismic signals alone were sufficient to localize, but the combination of acoustic and seismic data did not provide additional information using our probabilistic framework. This suggests that deploying seismic sensors can be sufficient to localize elephant rumbles using either approach set out here, with no added advantage for localization in having multi-modal systems. The underlying reasons for the less accurate combination approach may stem from the fact that each unimodal method inherits errors by: (i) timing measurement errors by all receivers and their synchronizing clocks, with corresponding implications for the cross-correlation step; (ii) numerical errors in the calculation of TDOAs; and (iii) estimating a constant wavespeed. The proposed joint method independently estimates wavespeeds for both types of wave, uses data from all four acoustic and all four seismic stations and calculates TDOAs for each method separately. This method is consequently prone to more sources of error, which ultimately could contribute to the larger residual estimates.

Our results also suggest that, on average, seismic methods outperform purely acoustic and joint methods, at least given our data selection and processing. This may suggest an advantage to deploying seismic over acoustic or multi-modal sensors for the purpose of localizing elephants from their rumbles. Given our inclusion criteria for rumble data, this was specifically in the cases where signals show a clearly visible spectrogram pattern upon manual assessment on both instrument types. This can be explained by the differences in the spectrograms between seismic and acoustic recordings. Our data show single fundamental vocalization frequencies (no harmonics) in the seismic spectrum, and multiple harmonics for co-generated acoustic vocalizations. This introduces ambiguity in the correlation of spectrograms in the acoustic data as different frequency content propagates at different speeds (dispersion), which possibly leads to the larger errors in the localization framework for the acoustic data. The spectrogram differences arise from differences in wave propagation between the air and ground, where the seismic wave attenuates higher frequencies faster (i.e. the ground acts as a low pass filter) [49]. This is a general effect and so we would expect the advantage in accuracy from seismic compared with acoustic recording to generally apply to other locations as well.

The relative performance of seismic data compared with acoustic data may, at first glimpse, appear surprising, given that the methods rely on an assumption of a single average wavespeed in the propagating medium. This is often contravened with seismic waves since the terrain is so variable [50]. Despite a complex terrain around the four stations, the localization performance of seismic recordings suggests that the heterogeneity of the seismic wavespeed might not be such a limiting factor over these spatial scales (for our data inclusion criteria).

Our data inclusion criteria meant that only nine events were selected for analysis, as they required discernible signals on all four instrument locations and both types. So, how could the number of rumbles successfully localized be increased and the method be made more robust to changing conditions? For the three-week duration of this work, we have found 38 events that were detected by the four acoustic sensors (but not by all four seismic sensors) and 29 that were detected by the four seismic sensors (but not by all four acoustic sensors). Many more rumbles were recorded on individual stations, but the complexity of the site used for recording may have constrained this because of its influence on wave amplitude and propagation distance; the site chosen for our recordings was a complex propagation terrain, crowded with continuous seismic and acoustic sources, meaning that many of them were overlapping, i.e. multiple rumbles from different animals arriving at one station at the same time, or they were not visible at one station or more. Therefore, only a subset of the recordings met our strict criteria. The terrain included unconsolidated sand, water, wet mud and a dam with significant local topography, which could limit the propagation distances of other calls so that they were not discernible at all four stations. Being the sole source of water over a large terrain, it attracted many species, and upwards of hundreds of elephants simultaneously. Indeed, seismic and acoustic noise levels are higher during the day than at night at the dam, and some of our equipment was not recording for some nights. It was also a partially windy location, introducing other forms of acoustic and seismic noise. All these factors contributed to this being a challenging locality in terms of signal versus noise. In other terrains, seismic or acoustic signals may well be a lot cleaner, and amplitudes clearly discernible over much greater distances [9], but indeed the reverse could also be true. For this method to have useful field applications, the method will need to locate more instances of rumbles in these complex locations. Given the promising results we have presented here, localization algorithms working with a mixture of acoustic and seismic recordings or with a smaller number of signals, rather than requiring all four acoustic or all four seismic signals to be present, could be developed.

Furthermore, chosen instruments generally play a role in the hit count for signal-to-noise ratios: in this study, we compared an off-the-shelf broadband seismic sensor with three components with an off-the-shelf acoustic sensor for listening to low-frequency (infrasound) vocalizations. The acoustic sensors used in this study show low sensitivity for frequencies between 20 and 40 Hz. Acoustic localization is, therefore, mostly based on harmonics, i.e. *f*_1+_, whereas seismic correlation/localization is almost exclusively based on the fundamental frequency, *f*_0_. Specifically designed acoustic sensors can possibly beat seismic sensors in terms of localization accuracy, if they could record the fundamental frequency and its harmonics of the rumble vocalization with a larger sensitivity and thus, once corrected for phase/group dispersal through pulse compression, obtain a sharper correlation peak. As such, one cannot extrapolate our findings to arbitrary settings or instrumentation, but being located in a challenging noise environment using typical instrumentation it is likely to apply to other scenarios as well. We suggest that future work should investigate the localization capabilities of infrasonic acoustic sensors and assess how sensor types and sensitivity ranges influence the localization accuracy. We believe that other environmental factors such as wind and other weather effects, topography and biotic or anthropogenic acoustic noise could also hamper infrasonic propagation and seismic equipment could still stand the test as a complementary tool for (infrasonic) wildlife monitoring.

The use of multiple sensors with a denser array covering the same overall area would also increase the number of elephant rumbles successfully localized as it would increase the signal-to-noise ratio, decrease propagation distances and make the method more robust to different locations and changing abiotic and biotic conditions. Furthermore, localization algorithms could benefit from varying wavespeeds, depending on the type of ground and direction of propagation, or full three-dimensional wave propagation models, taking into account variations in geological structure and topography [51].

Our results have implications for understanding how elephants may be using the seismic and acoustic components of their rumbles to transfer information. Firstly, detection of either acoustic or seismic TDOAs, in theory, would allow the elephant to localize an elephant rumble. For acoustic and seismic estimated wavespeeds of 350 m s^−1^ and 400 m s^−1^ and an interaural distance of 1 m, differences in arrival times would be 2.86 ms and 2.50 ms, respectively. Note that, for these wavespeeds on this terrain, it is unlikely that elephants can use the TDOAs between seismic and acoustic waves to orientate, as previously proposed [11], since the wavespeeds are very similar (but it may be more likely on geological structures with slower wavespeeds such as unconsolidated sand) [9]. Yet, localization may be easier for elephants using seismic waves in cases when both signals are discernible because of the increased frequency filtering, decreased dispersion and less ambiguity in correlating signals between ears. This suggests that using IPD (TDOAs) for localization will be easier using the seismic component of a rumble. Although it has been shown that elephants respond to seismic vocalizations [6], with detection using the feet or ears, the underlying psycho-acoustic process is yet to be confirmed. The differences in filtering during propagation between seismic and acoustic components will have important implications for long-distance communication in elephants [52,53]. This is an exciting avenue for future research, which will rely on methods to accurately locate the source of the rumble, as we have outlined here.

To successfully localize rumbles in the natural context, the elephants will need to cope with changing signal-to-noise ratios as physical (propagation distances, wind, geology, topology, vegetation) and biological (properties of rumble, other animals) factors vary. One way to cope with this would be to use either or both modes for locational information as external conditions change that favour the detection of one mode over another. Equally, elephants may repeat calls or choose to call under lower noise conditions in order to maximize the likelihood of localization by receivers, and receivers could use behavioural adaptations such as head scanning, turning, freezing and/or leaning to aid in signal detection and localization [8]. Further research shall give detailed insights into how elephants cope with the physical variability of their large and harsh terrains to promote information transfer, including the relative importance of the acoustic and seismic components of rumbles.

We propose that localizing elephant rumbles using seismic recordings has promising potential as a practical tool for research and conservation. The current system acts as a datalogger for passive acoustic monitoring, taking less than 20 s on a single CPU (Intel i5-9400F). Expanding our framework to real-time capabilities would require local event detection (e.g. to classify rumbles) and wirelessly communicating onset times to a central server. We plan to investigate this promising avenue in future research.

Beyond localization, this near real-time monitoring approach could be expanded to categorizing sources, thus locating particular animals or behaviours of interest, such as detecting alarm rumbles in elephants. It has been suggested that rumbles contain information on the individual animal and its emotional state [1,2,54]. Localization and classification approaches could, therefore, be used to monitor individual elephants, or entire herds, and their welfare across a spatially constrained area using dense arrays. Reliable detection of alarm behaviour in elephants in near real time could help in detecting poaching threats or mitigate human–elephant conflict by warning settlements that elephants are in their proximity [55–57]. In addition to the warning, tools could be developed to deter elephants when coming too close to human settlements, such as playing back sounds that represent threats to the animals (acoustically and/or seismically); however, negative stimulus reinforcement would be required to avoid habituation.

Beyond elephants, recent developments in passive acoustic monitoring, the method of studying wildlife using acoustic recordings, show promising results in the detection and classification of animal vocalizations using deep learning and supervised training on labelled data [58], an approach which also lends itself to seismic recordings [59]. Our data showed a cacophony of biological (and anthropogenic) sounds, including the steps of hoofed animals such as the Nubian giraffe (*Giraffa camelopardalis camelopardalis*), which is critically endangered, and the Grévy’s zebra (*Equus grevyi*), which is endangered. Remote localization and monitoring using their seismic ‘footprints’ could contribute to conservation of those species, a better understanding of group behaviour and ecological interactions without the need for invasive tagging of wildlife.

In conclusion, we have revealed the locational information present in the seismic components of elephant rumbles, where the localization quality of the signal renders it likely that this plays a role in elephant social behaviour, despite the possible limitations that the terrain poses as the propagation medium. We showed that seismic localization has better accuracy than acoustic localization using data from the same event for most of our chosen dataset (six/seven out of nine events, depending on the algorithm). This also brings to light the possibility of seismic wildlife monitoring, which has several advantages over other methods such as GPS tracking or camera trap surveillance: seismic sensors are easy to bury whereas acoustic microphones have to be placed above ground and are possibly exposed to strong winds, rain and solar radiation and damage by wildlife. Furthermore, they are omnidirectional and immune to thick vegetation, both of which impact camera traps. Practical approaches that allow non-invasive remote monitoring of animals will become increasingly important in the context of ongoing climate change, habitat loss and human–wildlife conflict, to better understand and protect biodiversity.

## Data accessibility

Additional details on data processing and localization for all events are in the electronic supplementary material, methods and figures S1–13. All code and data are available from https://github.com/mchlrnwld/Seismic-Rumble-Localisation (doi:10.5281/zenodo.4642565).
